# Soil microbiomes with distinct assemblies through vertical soil profiles drive the cycling of multiple nutrients in reforested ecosystems

**DOI:** 10.1186/s40168-018-0526-0

**Published:** 2018-08-21

**Authors:** Shuo Jiao, Weimin Chen, Jieli Wang, Nini Du, Qiaoping Li, Gehong Wei

**Affiliations:** 1 State Key Laboratory of Crop Stress Biology in Arid Areas, College of Life Sciences, Northwest A&F University, Yangling, 712100 Shaanxi People’s Republic of China; 2 College of Urban and Environmental Sciences, Peking University, Beijing, 100871 People’s Republic of China

**Keywords:** Soil microbiome, Reforestation, Vertical spatial variation, Multi-nutrient cycling

## Abstract

**Background:**

Soil microbiomes play an important role in the services and functioning of terrestrial ecosystems. However, little is known of their vertical responses to restoration process and their contributions to soil nutrient cycling in the subsurface profiles. Here, we investigated the community assembly of soil bacteria, archaea, and fungi along vertical (i.e., soil depths of 0–300 cm) and horizontal (i.e., distance from trees of 30–90 cm) profiles in a chronosequence of reforestation sites that represent over 30 years of restoration.

**Results:**

In the superficial layers (0–80 cm), bacterial and fungal diversity decreased, whereas archaeal diversity increased with increasing soil depth. As reforestation proceeded over time, the vertical spatial variation in bacterial communities decreased, while that in archaeal and fungal communities increased. Vertical distributions of the soil microbiomes were more related to the variation in soil properties, while their horizontal distributions may be driven by a gradient effect of roots extending from the tree. Bacterial and archaeal beta-diversity were strongly related to multi-nutrient cycling in the soil, respectively, playing major roles in deep and superficial layers.

**Conclusions:**

Taken together, these results reveal a new perspective on the vertical and horizontal spatial variation in soil microbiomes at the fine scale of single trees. Distinct response patterns underpinned the contributions of soil bacteria, archaea, and fungi as a function of subsurface nutrient cycling during the reforestation of ex-arable land.

**Electronic supplementary material:**

The online version of this article (10.1186/s40168-018-0526-0) contains supplementary material, which is available to authorized users.

## Background

With their intensified use by humans, ecosystems are facing biodiversity losses and changes in their ecosystem functioning and services [1–5]. Among the most serious issues is agricultural intensification, which is considered a major threat to global biodiversity [6]. Increasing concerns have been raised, because agricultural intensification could adversely influence natural environments in many ways, including large-scale soil degradation, loss of productivity, increased greenhouse gas emissions, accumulation of pesticides, and diminished availability and quality of water [1, 7]. Reforestation of arable land represents one of the most widely used restoration strategies, one that could restore natural ecosystem functioning and soil properties, but also influence belowground microbial community dynamics [8]. Since soil microorganisms are major component of terrestrial ecosystems, it is of fundamental importance to determine the temporal changes in their community dynamics, as well as their contributions to soil ecological restoration, during the long-term restoration process of natural ecosystems.

Increasing attention has focused on the significance of soil microbiomes with extremely complex drivers, as a combination of bacteria, archaea, and fungi [8, 9]. Soil microbiomes play important roles in ecosystem functioning, such as by participating in the biogeochemical cycling of soil nutrients [10, 11]; acting as decomposers, mutualists, or pathogens to influence the growth of macro-organisms [12]; and emitting greenhouse gases that may accelerate global climate change [13]. Numerous studies have focused exclusively on the top 20 cm of the soil column or less, in which the microbial biomass, activity, and diversity are the greatest [14]. Nevertheless, with its large volume throughout the depth of the soil profile, the subsurface soil (i.e., deeper than 20 cm) contains nearly 35% of the total microbial biomass and also harbors diverse microbes [15, 16]. In particular, the huge reservoirs of subsurface soil microbiomes can play potentially important roles in soil formation, pollutant biodegradation, and groundwater quality maintenance [14–17]. Distinct microbial community structures have been observed between the surface and subsurface soils because of their different environments, for which microbial diversity varied with soil depth [16–19]. However, these studies focused on a certain microbial kingdom (i.e., bacteria or fungi) and the soil depth had a range of 0–100 cm. In contrast, little is known about how subsurface soil microbiomes and properties respond to the restoration process of natural ecosystems, especially in the deeper soil profiles (i.e., 100–300 cm).

Microbial distributions have been well investigated at global [10], continental [20], and regional scales [21, 22]. Key environmental factors, such as soil pH, available nutrients, soil texture, and climatic conditions, can all significantly affect microbial community distributions [23, 24]. Unlike macro-organisms, however, microbes with an extremely small size could partition more niches at a much finer scale, for example, at the centimeter level [25]. Surrounding a tree, soil microbiomes might be influenced by an inconsistent environmental heterogeneity gradient along the radial distance of the tree’s root system, mainly through the release of exudates and mucilage. Yet our understanding of how soil microbiomes are distributed on a fine scale with respect to this plant root-associated gradient remains surprisingly limited, especially since the kind and integrity of ecosystem services will depend on the ecological functioning of local organisms [26].

Generally, an ecosystem can perform multiple functions and services, and how this ecosystem multifunctionality is linked to local biodiversity has been researched in the past two decades, primarily in plants [27–29]. Recent studies demonstrated that plants could enrich soil microbes with evolved genes that adapt to plant environments [30], which may influence ecosystem multifunctionality belowground [29]. Plant roots are known to release exudates and mucilage into their surrounding environments, which often shape the associated soil microbial communities [31, 32]. Moreover, these plant-associated microbes are capable of influencing many critical ecosystem functions, such as nutrient acquisition by plants and the cycling of resources between above- and belowground communities [33, 34]. Previous work has shown soil microbial diversity is a key driver of multifunctionality in terrestrial ecosystems [35, 36]. However, it remains unclear what contribution different microbial groups make to the cycling of multiple nutrients in subsurface ecosystems, especially during ecological restoration of ex-arable land.

The aim of this study was to investigate the vertical assembly of soil microbiomes at a fine scale and their contribution to soil multi-nutrient cycling in the subsurface profiles during the successional development of restored soil ecosystems. We used a well-established chronosequence of reforestation sites on ex-arable, formerly cultivated, lands that represent over 30 years of nature restoration. The biodiversity of soil archaea, bacteria, and fungi were determined through soil depths of 0–300 cm, and at distances of 30–90 cm from a single plant, for a nature restoration process occurring over a 30-year period. Our study could provide an integrated perspective on vertical responses of soil microbiomes to reforestation at a fine spatial scale and further suggest their important roles in soil nutrient cycling, particularly in the subsurface of terrestrial ecosystems.

## Results

### Vertical variation in soil properties during reforestation of the ex-arable land

In the course of reforestation of the ex-arable land, the soil properties typically changed along the chronosequence (Additional file 1: Figure S1A). Available phosphorus (AP) significantly decreased going from arable land to the 30-year reforested soil, while both pH and organic matter (OM) were similar between the arable land and reforested soils. Nevertheless, the reforested soils contained significantly higher nitrate nitrogen (NO_3_^−^-N) and available potassium (AK) than did the arable land, and total phosphorus (TP) peaked in the 10-year reforested soil.

Regarding the soil profiles, we found that the soil properties had different variation with depth between the superficial (0–80 cm) and deep layers (100–300 cm; Additional file 1: Figure S1B). As soil depth increased, pH significantly increased in the superficial layers, while it slightly decreased in the deep layers. OM and NO_3_^−^-N significantly decreased in the superficial layers but did not change in the deep layers. AK significantly decreased in superficial layers, whereas it increased in the deep layers. Interestingly, AP increased significantly with increasing soil depth through the entire profiles. Only AP presented a differing variation trend; its concentration first decreased and then increased in arable land, while it consistently increased through the entire profiles of the reforested soils (Additional file 1: Figure S2).

Next, we estimated the variation in all soil properties between the arable land and reforested soils. The pH and AP were significantly lower in the superficial layers than in the deep layers, while OM and NO_3_^−^-N had a reversed pattern (Additional file 1: Figure S3). We did not find any significant differences among the different radii distances-from-tree samples in the reforested soils. Given the clear and distinct variation patterns of the soil properties, this division of superficial versus deep layers for soil depths was adopted in the subsequent analyses.

### Temporal and spatial distribution patterns of soil microbiomes at fine scale

Across all the samples, we obtained a total of 18,852,624, 18,631,178, and 22,214,132 high-quality bacterial, archaeal, and fungal sequences, which were respectively grouped into 17,687, 10,892, and 17,347 OTUs when using the 97% sequence similarity cutoff. Bacterial sequences were primarily composed of the phyla Proteobacteria (28.9%), Actinobacteria (19.0%), Acidobacteria (16.7%), Chloroflexi (8.7%), and Nitrospirae (5.8%). The majority of archaeal sequences belonged to the phyla Thaumarchaeota (53.8%) and Euryarchaeota (3.5%). The most abundant fungal phyla were Ascomycota (48.7%), Basidiomycota (44.1%), and Zygomycota (5.4%).

**Fig. 1:**
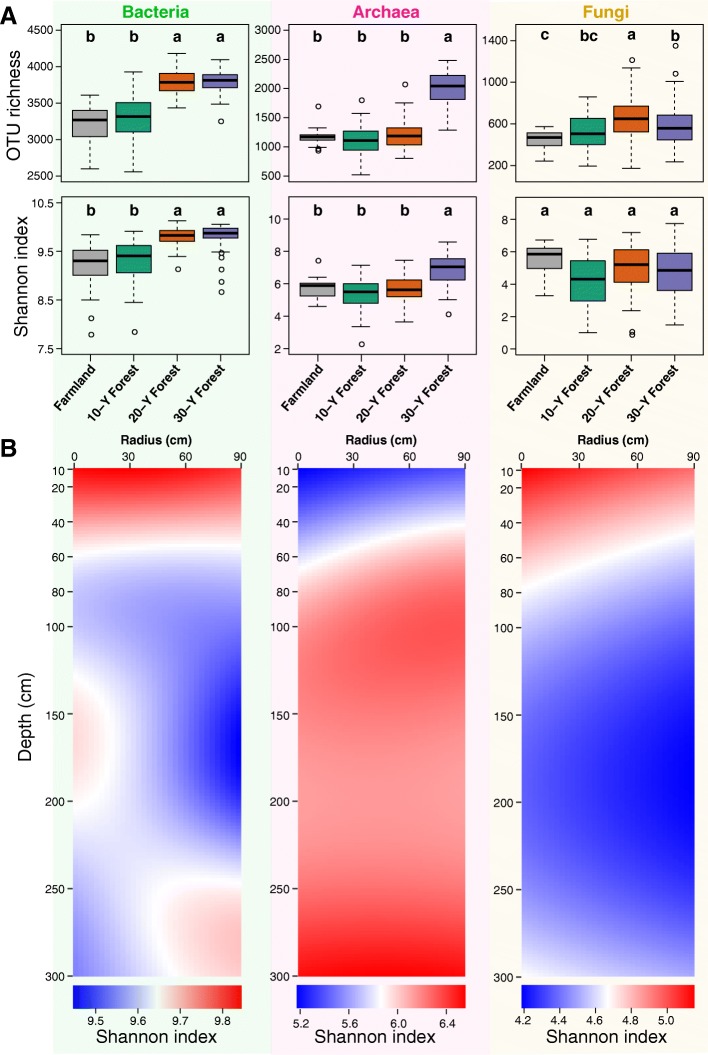
General patterns of microbial alpha-diversity during reforestation of the ex-arable land and at a fine scale of single trees. **a** Variation in the alpha-diversity of soil bacteria, archaea, and fungi during reforestation of ex-arable land were estimated via linear mixed-effects models, with samples from the same tree (reforested soils) or same core (arable land) set as random effects. Boxplots that do not share a letter are significantly different (*P* < 0.05). **b** Vertical and horizontal spatial distribution of Shannon index for bacterial, archaeal, and fungal communities around the tree in reforested soils. The intensity of the color from blue to red is proportional to the value of Shannon index from small to large

Alpha-diversity levels of soil bacteria, archaea, and fungi were all higher in the reforested soils than in arable land, except the Shannon index for fungi (Fig. 1a). In the reforested soils, bacterial and fungal diversity decreased, whereas archaeal diversity increased with increasing soil depth in the superficial layers (Fig. 1b). The significance of these trends was confirmed by least-squares linear regression analysis (Additional file 1: Figure S4).

Furthermore, distinct variation trends in alpha-diversity were observed between the arable land and reforested soils (Additional file 1: Figure S4). For bacteria, the alpha-diversity indices decreased as soil depth increased in the arable land soils; in the forested soils, however, this trend only occurred in the superficial layers. For fungi, the alpha-diversity indices in arable land soil did not change along the soil depth profile; the indices all increased first and then decreased with depth in the reforested soils. The differences in the alpha-diversity indices between the superficial and deep layers were confirmed via the Wilcoxon rank sum test (Additional file 1: Figure S5). We found that the bacterial diversities of arable land were significantly higher in superficial than deep layers, which was not observed in reforested soils, while archaeal and fungal diversities had the reversed pattern.

**Fig. 2:**
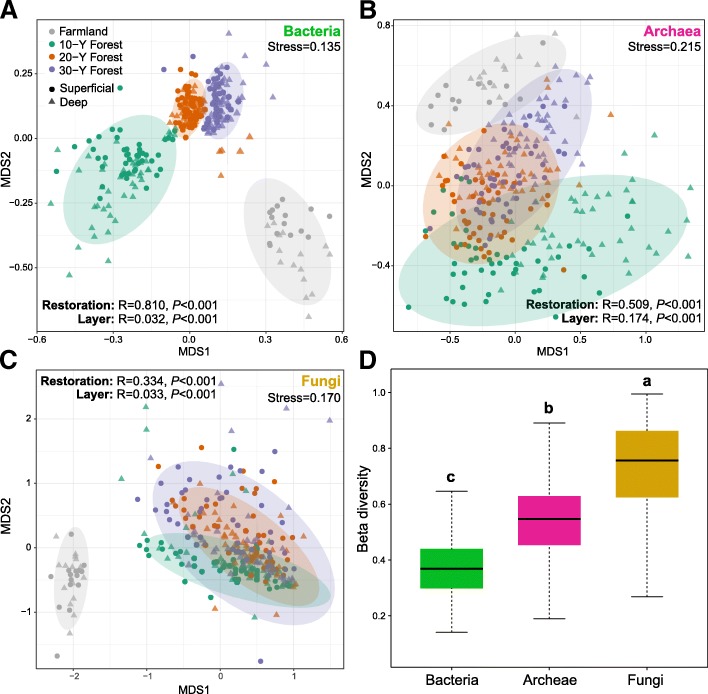
General patterns of microbial beta-diversity in superficial and deep soils during reforestation of the ex-arable land. NMDS showed the structure of microbial community for soil bacteria (**a**), archaea (**b**), and fungi (**c**). 95% confidence ellipses were shown around the samples grouped based on reforestation of the ex-arable land. Similarity values among the samples during reforestation of the ex-arable land (“restoration”) and between superficial and deep layers (“layer”) were examined via the ANOSIM test, which are shown in each plot. (**d**) Differences in beta-diversity among the bacteria, archaea, and fungi were estimated based on a Bray–Curtis distance matrix of all 300 soil samples, including 44,850 data points for each microbial group. Data that do not share a letter are significantly different between treatments (*P* < 0.05; multiple comparison with Kruskal-Wallis tests)

Non-metric multidimensional scaling (NMDS) analysis revealed that the soil samples of arable land and different reforested years formed distinct clusters in the ordination space (Fig. 2a–c), with significant differences being found at taxonomic levels (ANOSIM test). These differences among arable land and reforested soils were the largest for bacterial communities, followed by archaeal and fungal communities; this indicates that soil bacterial communities were more influenced by the reforestation of the ex-arable land. In addition, we observed significant differences in microbial community between superficial and deep layers. These differences were larger for archaeal communities than bacterial and fungal communities, suggesting that archaeal communities were more sensitive to soil depths. Furthermore, we estimated the differences in beta-diversity among different microbial community groups based on Bray–Curtis distance (Fig. 2d). Fungal communities showed the highest beta-diversity, indicating their higher dispersion.

**Fig. 3:**
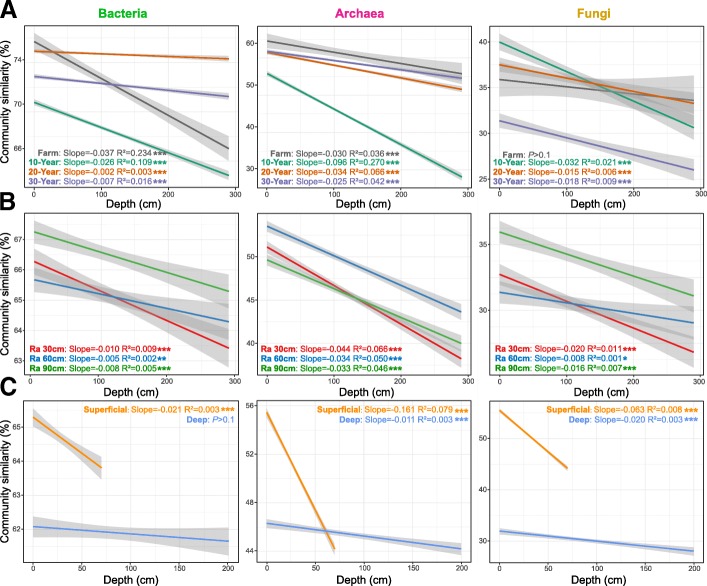
Similarity of soil bacterial, archaeal, and fungal communities between arable land and reforested soils (**a**), radii around the tree (**b**), and soil layers (**c**). Community similarity was calculated based on 1—[dissimilarity of the Bray–Curtis distance metric]. The lines denote the least-squares linear regressions across soil depth, with their 95% confidence intervals (gray-shaded areas). *P < 0.05; **P < 0.01; ***P < 0.001

The vertical spatial variation in each microbial community group down through the soil depth profiles were compared between the arable land and reforested soils, the superficial and deep layers, and among the reforestation years and radii around the tree. In the course of reforestation, the vertical spatial decay relationship (VDR) slopes of all the microbial groups were steepest in the 10-year reforested soil, though basically similar between the 20-year and 30-year reforested soils (Fig. 3a). Particularly, the significant VDR slopes of bacterial communities increased from arable land to reforested soils; the archaeal VDR slope of arable land was larger than 10-year and 20-year reforested soils; the non-significant fungal VDR for arable land turned to be significant in the course of reforestation. These indicate that the reforestation of the ex-arable land deceased vertical spatial variation in bacterial communities, but increased the variation in archaeal and fungal communities. These observations were also confirmed by the different tests of microbial beta-diversities between superficial and deep layers (Additional file 1: Table S2). With regard to radii around the tree, the slopes of all microbial groups were steepest for soils at a 30-cm distance from the tree (Fig. 3b). In addition, soil microbiomes in the superficial layers showed much steeper slopes of VDRs than those in the deep layers (Fig. 3c). Interestingly, VDR slopes of archaea were the steepest, followed by those of fungi and bacteria groups.

**Fig. 4:**
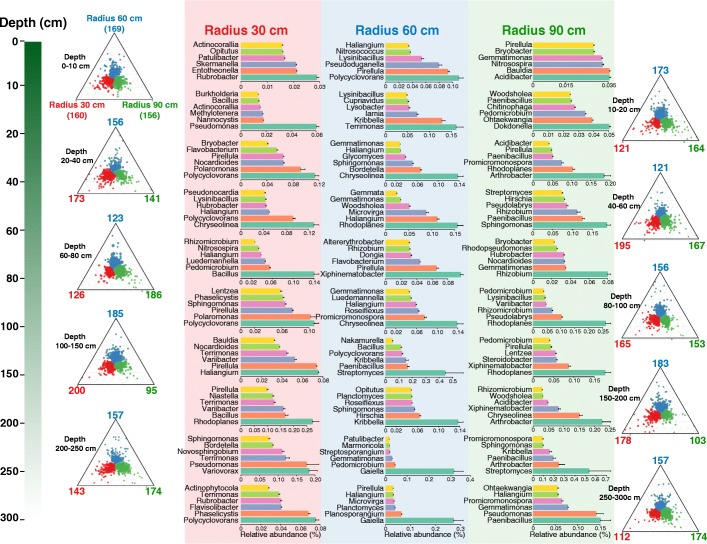
Taxonomic distribution of bacterial taxa responsible for community differentiation among different radii to the tree at each soil depth. The most abundant six genera are displayed in barplots. Ternary plots show the distributions of these differentiation taxa. Each circle represents one OTU. The size of each circle represents its relative abundance. The position of each circle is determined by the contribution of the indicated compartments to the total relative abundance. The colors of circles mark the OTUs significantly enriched among different radiations to plant (false discovery rate < 0.01). The numbers of differentiate OTUs are displayed at the vertex of the ternary plots

To identify those microbial taxa responsible for community differentiation among radii around the tree, we used a linear model analysis to determine the indicator OTUs for each radius (i.e., distance-to-tree) group for each soil depth (Fig. 4 and Additional file 1: Figure S11). In general, the identified indicator OTUs were distinct among the different depth layers, although they were situated at a very fine scale. Arguably, the distributions of these indicator OTUs were complex; nonetheless, we did obtain some interesting results. For example, the OTUs belonging *Polycyclovorans* were mainly abundant in soils at 30-cm and 60-cm radii; *Bacillus* were dominant in soil taken from the 30-cm radius; *Gaiella* were more abundant in the deeper-layer soil (i.e., 200–300 cm) taken at 60-cm radius; *Paenibacillus* and *Acidibacter* were significant indicators for soils occurring at 90-cm radius; *Rhizobium* were more abundant at radii of 60 cm and 90 cm in soils from a depth of 60–80 cm (Fig. 4). Concerning the fungi, *Lysurus* were indicators for soil from the depth of 0–10 cm of 30-cm radius, while dominant for 90-cm radius soils at depths of 20–80 cm, and more abundant for 60-cm radius soils at depths of 80–200 cm. Indicator OTUs belonging to *Fusarium* were mainly observed for soils taken at 30-cm and 90-cm radii, throughout the profiles. Detailed descriptions are provided in Additional file 1, which also contains information on the temporal and spatial distribution patterns of dominant microbial taxa.

### Potential drivers of soil multi-nutrient cycling in reforested ecosystems

**Fig. 5:**
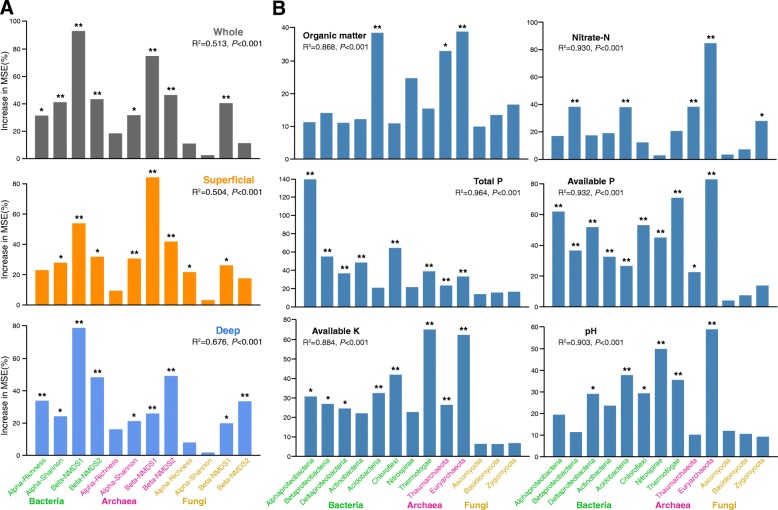
Potential drivers of variation in soil multi-nutrient cycling in reforested ecosystems. **a** Random forest (RF) mean predictor importance (percentage of increase of mean square error) of microbial alpha- and beta-diversity indices as drivers for the soil multi-nutrient cycling index, in the whole profile, and superficial and deep layers separately. **b** RF mean predictor importance (percentage of increase of mean square error) of dominant phyla (> 5% of total community) as drivers for soil properties in the whole profile. The accuracy importance measure was computed for each tree and averaged over the forest (5000 trees). Percentage increases in the MSE (mean squared error) of variables were used to estimate the importance of these predictors, and higher MSE% values imply more important predictors. Significance levels are as follows: *P < 0.05 and **P < 0.01. MSE, mean squared error

To disentangle the potential main drivers of soil nutrient cycling in reforested ecosystems, we identified the main microbial predictors for the soil multi-nutrient cycling index by random forest (RF) analysis (Fig. 5a). Bacterial beta-diversity was found to be the most important variable for predicting the soil multi-nutrient cycling index throughout the vertical soil profiles followed by archaeal beta-diversity. Comparing soil depths, the microbial diversity indices which were associated with the variations in soil multi-nutrient cycling index differed between superficial and deep soil layers. While archaeal beta-diversity best predicted these dynamics in superficial layers, bacterial beta-diversity was instead pivotal in deep layers.

We also evaluated the biological contributions of dominant microbial phyla to soil properties via a RF analysis (Fig. 5b). Evidently, not all microbial phyla contributed alike to the various edaphic variables. For example, Euryarchaeota was the most important variable for predicting many soil properties, including OM, NO_3_^−^-N, AP AK, and pH (*P*s < 0.01), indicating its importance in soil nutrient cycling during reforestation. Other important variables for predicting soil properties were the Acidobacteria, for pH and OM (*P*s < 0.01); the Alphaprobeobacteria, for TP and AP (*P*s < 0.01); the Thermotogae, for AP and AK (*P*s < 0.01); and the Thaumarchaeota, for OM and NO_3_^−^-N (*P* < 0.01). With regard to fungi, only the Zygomycota contributed a little for NO_3_^−^-N (*P* < 0.05). These observations were supported by the results of multivariate regression analysis (Additional file 1: Tables S3 and S4).

## Discussion

In this study, we found that distinct responses of soil microbiomes to reforestation drove potential multi-nutrient cycling in vertical soil profiles. Most importantly, our results reveal that archaeal beta-diversity played a major role in soil multi-nutrient cycling in the superficial layers, while bacterial beta-diversity contributed most in deep layers. This study sheds light on the vertical and horizontal spatial variation in soil microbiomes at the fine scale of single trees.

Based on the vertical variation we found in the soil properties, the soil depths in the present study could be divided into two distinguishable layers: a superficial (0–80 cm) and a deep layer (100–300 cm), which may correspond to the mineral soil horizons (A and B horizons) and deeper saprolite (C horizon) [37], respectively. The soil properties showed greater variation in the superficial than in the deep layers, which might be related to the high microbial activity and high biomass of plant roots and more anthropogenic disturbances occurring in the upper soils [14]. Previous work has demonstrated that the diversity of bacteria typically decreases with increasing soil depth [16]. Our results go further, showing that bacterial and fungal diversity decreased, whereas archaeal diversity increased, with increasing soil depth in the superficial layer. This may be partially explained if oxygen also decreased with soil depth, since these microbiomes prefer different oxygen conditions—archaea are mainly anaerobic, while bacterial and fungi are mainly aerobic [38, 39]. Additionally, we found greater variation in beta-diversity for soil microbiomes in the superficial than in the deep layers, which could be related to the former’s enhanced vertical variation in soil properties.

Our results showed that the reforestation of ex-arable soils increased the biodiversity of soil microbiomes and shaped their structure, highlighting the vital importance of soil restoration [40, 41]. Prior work done at our site investigated soil microbial responses to reforestation, finding that after 25 years, it had rapidly altered the soil fungal community composition and changed bacterial community composition [42]. Yet, here we found that soil bacterial communities were instead influenced more by the reforestation of ex-arable land than were fungal communities. This contrasting pattern could reflect divergent microbial responses in the surface and subsurface soils since their environmental conditions also vary with soil depth [16–19]. Indeed, our study focused primarily on the vertical assembly of soil microbiomes through soil profiles during the successional development of restored ecosystems. These temporal dynamics, however, were inferred from a chronosequence of reforestation sites. Unlike microbial dynamics studied at a fine temporal scale (e.g., month or year) [43–45], chronosequence studies generally focus on succession over several decades, or even hundreds of years, with sampling often done at multiple chronosequence sites but at a single time point [42, 46, 47]. The few studies assessing successional patterns of microbial communities along environmental chronosequences have focused on undisturbed salt marshes [46, 47], receding glacier forelands [48], and abandoned agricultural fields [42]. Chronosequences, which presume a space-for-time substitution, thus offer unique opportunities to investigate the dynamic assembly of soil microbiomes during the successional development of restored ecosystems.

In this study, we found that reforestation drove distinct vertical responses of soil microbiomes at the fine scale of single trees. The survival and activity of microbes is often limited in many soils, generally encompassing a wide range of environments [37]. Various microbial groups prefer different growth conditions, with substantial differences in their habit and dispersal capability [38, 49]. Herein, we found that reforestation reduced the vertical spatial variation in bacterial communities, but increased the variation in archaeal and fungal communities. During reforestation, plants can reduce the available niche heterogeneity by homogenizing local carbon availability, pH, and water among the soil microsites, thus generating less spatial heterogeneity through the soil profiles [50, 51]. One study found that reforestation could modify soil pH if the tree species and initial pH are properly matched [52]. Due to their relatively high intrinsic growth rates, bacteria are generally more resilient in the face of disturbances and perturbations [53], so they could more rapidly respond to the environmental filtering induced by reforestation. This also explained that bacterial communities were more influenced by the reforestation of the ex-arable land. Deep tillage of arable land might expose its subsoil to air; since archaea prefer low oxygen conditions [18, 38], they likely were more sensitive to soil depth in the reforested soils having reduced land-use intensity. The community of soil fungi with filamentous growth could exhibit antagonistic interactions due to dispersal limitation [49, 54], resulting in distinct vertical distributions; it might be stronger under undisturbed conditions.

Furthermore, we observed steeper VDR slopes of all microbial groups for the 10-year compared with 20-year or 30-year reforested soils, which indicates a gradient effect driven by forest establishment and growth. Plants could influence soil microbiomes directly through the provision of carbon compounds—including root exudates, mucilage, and plant litter—or via secondary metabolites, which have been found to be closely associated with succession in terms of plant growth [55, 56]. However, in the present study, the impacts of the reforestation process were not restricted to the superficial layer, but extended into the deep layers. For example, distinct vertical distributions of some dominant phyla were observed between the superficial and deep layers. Previous work has demonstrated that niche filtering is more important for microbial community selection in the rhizosphere soil than in bulk soil, due to the potential interactions of soil physicochemical characteristics and root-derived products [57, 58]. These discoveries could be important for fully describing the ecology of soil microbiomes belowground, and for understanding their vertical distribution and assembly in deep soil layers of terrestrial ecosystems, especially during the reforestation of ex-arable land. This type of land use change is increasingly popular, with projects primarily designated for wood production, soil and water conservation, and increasing carbon storage and mitigating climate change [59]. Our findings thus provide an integrated microbial perspective of vertical responses of soil microbiomes to reforestation, suggesting reforestation strategies and policies should give due consideration to distinct community assembly and functions of microbial groups (e.g., archaea, bacteria, and fungi) through soil profiles.

Soil environments only centimeters apart could differ substantially in their abiotic characteristics, rates of microbial activity, and microbial community composition [37]. Bacterial communities near the plant root or fungal-hyphal networks may differ considerably from those found in “bulk” soils just a few centimeters away [60]. Our results revealed that, along with the substantial variation found in soil properties, the soil microbiomes followed a vertical distribution in terms of their diversity and community assembly. However, in the horizontal aspect, no significant differences in soil properties were observed among different distances (radii) to trees in the reforested sites. The radius slightly but significantly influenced the assembly of bacterial and archaeal communities but not the fungal community, which might be attributable to their different growth habits (unicellular bacteria and archaea versus and filamentous fungi) [49]. Nevertheless, we observed complex distributions of the identified indicator OTUs for each radius group through soil depth profiles. The VDR slopes were steepest for the 30 cm radius soils and the networks nearer to the trees were more connected and had closer relationships. These results suggest there is a discernible horizontal distribution of soil microbiomes with distance to a single tree. Our study also revealed that the belowground gradient effect of roots upon the soil microbiomes is related to their distance to a tree. Surrounding a tree, an environmental heterogeneity gradient could be generated along the radial axis of the root system due to the dispersal limitations of released root exudates and mucilage among the root zone soils. These closely linked root exudates can become augmented going from rhizosphere to bulk soils, which could act as substrates, or as chemotactic or signaling molecules to mediate the assembly of soil microbiomes [61–63]. Ramette and Tiedje [64] found that differences in the bacterial group *Burkholderia* at small scales were greater than those occurring on large spatial scales and that the surrounding environmental conditions contributed most to community assembly at the small scale.

Complex variation occurred in soil properties in the course of reforestation, such as increases in soil AK and NO_3_^−^-N and deceases in pH, OM, and AP. These changes could be partly explained by soil microorganisms themselves, whose activities have proven essential for the functioning of these nutrient cycles [10, 11]. Studies have shown that reforestation contributes to changed edaphic properties via root exudates, mucilage, and plant litter provided by trees [32, 65]. However, these environmental changes can also lead to an altered microbial community [66], whose microorganisms are vital engines that drive Earth’s biogeochemical cycles [11, 67]. A recent study investigating biotic and abiotic factors on the Tibetan Plateau demonstrated positive associations between aboveground and belowground biodiversity and ecosystem multifunctionality, which was mediated by climate [35]. Moreover, soil microbial diversity was directly and positively related to multifunctionality in terrestrial ecosystems, based on databases for 78 global drylands and 179 locations across Scotland [36]. Therefore, in the present study, we evaluated the microbial contributions to cycling of multiple nutrients in soil. From this, we reasonably inferred that bacterial and archaeal compositions were closely related to multi-nutrient cycling in the reforested subsurface ecosystems. Supporting this view is the exchange of geochemical resources in the terrestrial subsurface that was found to be driven by interactions among dominant members of the microbial community [68].

Bacteria are known to be involved in various soil processes and global biogeochemical cycling [11], such as organic matter degradation [43, 44] and nitrogen cycling [69]. In the deep-subsurface community, metabolic cooperation via syntrophy between bacterial groups plays a critical role in the survival of the whole community under oligotrophic conditions [70]. This might explain the large contribution of bacterial composition to multi-nutrient cycling in the deep soil. Archaea constitute a considerable fraction of the microbial biomass on Earth and have been found to contribute to the biogeochemical cycles of carbon and hydrogen metabolism [71]. Archaeal methanogenesis is typically considered the dominant process in anaerobic habitats [38]. In addition, archaea predominate among ammonia-oxidizing prokaryotes in soils [72]. Ammonia oxidation is the first step in nitrification, a key process in the global nitrogen cycle that results in the formation of nitrate through microbial activity [72]. In the present study, we found that Euryarchaeota was the most important variable for predicting many nutrient properties, including OM, NO_3_^−^-N, AP, and AK; this indicates archaea play potential roles in the biogeochemical cycling of multiple nutrients in the terrestrial subsurface. This observation might also imply a role of archaeal composition for predicting multi-nutrient cycling in the superficial soil, as the archaeal diversity increased with increasing soil depth only in superficial layers. Overall, our results demonstrate the crucial participation of soil microbiomes in soil nutrient cycling, notably in the subsurface of reforested ecosystems, whose fertility maintenance should figure prominently in ecological sustainability plans targeting multifunctionality for the better provision of key ecosystem services.

## Conclusion

In the present study, we quantified the dominant roles of archaeal and bacterial beta-diversity in the potential cycling of multi-nutrients in terrestrial surface and subsurface ecosystems during the course of reforestation of ex-arable land. The distinct vertical responses of soil microbiomes to reforestation could be important for fully describing the belowground soil ecology and for understanding their vertical distribution and assembly in deep soil layers of terrestrial ecosystems. We propose that a focused and novel framework for the study of specific roles of soil microbiomes in plant productivity and nutrient cycling at a fine scale is now necessary to appreciate and apply their contributions to key ecosystem functioning and services. It is paramount that such investigations evaluate both vertical and horizontal distributions during the successional development of restored terrestrial ecosystems.

## Methods

### Ex-arable land chronosequence

A chronosequence of reforestation sites on ex-arable lands that represent over 30 years of nature restoration was selected for use in this study. These sites are located in the Shaanxi Province and south of the Loess Plateau in China, where down to a 50-m depth the Loess soil is predominantly silt loam. This is a warm semi-humid temperate region with a continental monsoon climate. The mean air temperature and annual precipitation are 12.7 °C and 580 mm, respectively. The history of agricultural use spans more than 50 years, with crop rotations, including those of wheat and maize. The fields were separated and reforested from agricultural usage at different points in time: 10-year, 20-year, and 30-year forests have thus developed in different locations with their corresponding coordinates recorded (Additional file 1: Table S1). Black locust (*Robinia pseudoacacia*) is the dominant tree species at all the sites. An active area of arable land growing both wheat and maize was selected adjacent to the reforested sites.

### Sample collection

In the area of arable land, we selected three sampling points that were 500 m apart from each other. In the reforested area, three sites with different years (10, 20, and 30) of forest regrowth were selected. Within each site, three sampling points were chosen and three trees were randomly selected at each sampling point. Around each tree, five soil cores were taken at evenly distributed radii of 30-cm, 60-cm, and 90-cm distances to the trunk (i.e., 15 cores per tree). For each core, after first removing loose debris from the forest floor, soil subsamples were collected from a 300-cm-long vertical profile that corresponded to depths (cm) of 0–10, 10–20, 20–40, 40–60, 60–80, 80–100, 100–150, 150–200, 200–250, and 250–300. Each soil composite sample was a mixture of the five soil cores for a given depth layer at the same radius from the same tree. Then, for each sampling point, the composite samples from the three trees were pooled together for the same layer and radius. In total, 300 = 3 replications × 10 depths (arable land) + 3 replications × 3 radius × 10 depths × 3 ages (forest) soil samples were obtained. Visible plant roots, stones, litter, and debris were removed from each soil sample, which was then divided into two subsamples. One subsample was immediately stored at − 80 °C for the DNA analysis, and the other was air-dried for the physicochemical analysis. The physicochemical properties of all the soil samples were quantified as previously reported [73], including pH, OM, NO_3_^−^-N, AK, AP, and TP.

### DNA extraction, PCR, and high-throughput sequencing

Genomic DNA was extracted from 0.5-g soil samples by using the MP FastDNA spin kit for soil (MP Biomedicals, Solon, OH, USA) according to the manufacturer’s instructions. We amplified a region of the 16S rRNA gene, for archaea and bacteria, and a region of the ITS1 gene for fungi. The archaeal and bacterial 16S rRNA genes were amplified by the primer pairs Arch519F (CAGCCGCCGCGGTAA)/Arch915R (GTGCTCCCCCGCCAATTCCT), and 515F (GTGCCAGCMGCCGCGGTAA)/907R (CCGTCAATTCCTTTGAGTTT), respectively; the fungal ITS1 gene was amplified by primer pair ITS5-1737F (GGAAGTAAAAGTCGTAACAAGG)/ITS2-2043R (GCTGCGTTCTTCATCGATGC). All the samples were amplified in triplicate, and no-template controls were included in all steps of the process. Triplicate PCR amplicons were pooled together, after which they were detected by electrophoresis in a 2% (*w*/*v*) agarose gel. PCR products with a bright band were mixed in equal density ratios and purified with GeneJET Gel Extraction Kit (Thermo Scientific). The purified PCR amplicons products were sequenced on the Illumina MiSeq (300-bp paired-end reads) platform (Illumina Inc., San Diego, USA) at the Novogene Bioinformatics Technology Co., Ltd. (Beijing, China). The acquired sequences were filtered for quality according to previous work [74], and any chimeric sequences were removed with the USEARCH tool based on the UCHIME algorithm [75]. The sequences were split into groups according to their taxonomy and assigned to operational taxonomic units (OTUs) at a 3% dissimilarity level, by using the UPARSE pipeline [75]. Those OTUs with less than two sequences were removed, and their representative sequences were classified within the SILVA database release 128 for bacteria and archaea, and UNITE+INSD (UNITE and the International Nucleotide Sequence Databases) for fungi.

### Statistical analyses

All statistical analyses were conducted in the R environment (v3.2.2; http://www.r-project.org/). To assess the microbial diversity and abundance, the alpha (*α*) of OTU richness and Shannon-Wiener index were calculated, while the microbial beta-diversity was estimated according to the Bray–Curtis distance between the samples. Means of alpha-diversity for soil bacteria, archaea, and fungi during the reforestation of ex-arable land were compared via linear mixed-effects (LME) models, with samples from the same tree (reforest soils) or the same cores (arable land) considered as random effects, by using the function “lme” in the “nlme” package. The vertical VDRs were calculated as the linear least-squares regression relationships between soil depth and microbial community similarity (based on 1—[dissimilarity of the Bray–Curtis distance metric]). The linear least-squares regression relationships between the soil depth and soil properties, *α*-diversity and some dominant phyla were also estimated. The adjusted *R*^2^ value was considered as the criteria for selecting whether the models were fitted with the whole depths (300 cm) or with superficial (0–80 cm) and deep layers (100–300 cm) separately.

Canonical discriminant analysis (CDA) was used to identify the significant taxonomic differences associated with different years of the reforested soils, by using the “candisc” function of the “candisc” package. To identify the microbial taxa responsible for the community differentiation among the different tree radii, we employed linear statistics on all of the OTUs in each soil depth with the “limma” package. The differential OTUs with false discovery rate-corrected *P* values < 0.01 were identified as indicator OTUs, which were illustrated by ternary plots with the “ggtern” package. The taxonomic distribution of these indicator OTUs at each radius distance to the tree are displayed in bar-graphs for the most abundant six genera.

Ecosystems perform multiple simultaneous functions and services (multifunctionality), rather than a single measurable process. Multiple nutrient cycling is therefore the most important terrestrial ecosystem process for supporting human welfare [2]. To quantify this vital provision, we constructed a soil multi-nutrient cycling index—analogous to the widely used multifunctionality index—which included five measured nutrient properties: organic matter, nitrate nitrogen, total phosphorus, available phosphorus, and available potassium [35, 36]. These nutrient properties deliver some of the fundamental supporting and regulating ecosystem services [29, 35, 36]. For example, organic matter, nitrogen, and phosphorus are the nutrients that most frequently limit primary production in terrestrial ecosystems [76]. Nitrate is an important nitrogen source for both microorganisms and plants [76]. Available phosphorus is the main phosphorus source for plants and microorganisms, and it is linked to organic matter decomposition [76]. Potassium is the third essential macronutrient required by plants; it participates in a multitude of biological activities that maintain or improve crop growth, such as protein synthesis, enzyme activation, and photosynthesis [77].

To derive a quantitative soil multi-nutrient cycling index value for each site, we first normalized (log-transformed as needed) and standardized each of the five nutrients properties using the *Z* score transformation. These standardized ecosystem functions were then averaged to obtain this index [36]. We used this index to quantify soil multi-nutrient cycling because (1) it is a straightforward and interpretable measure of a community’s ability to sustain multiple functions at once and (2) we intended to explore the microbial contributions to service-based outcomes [29, 35, 36].

Microbial beta-diversity was quantified by using two axes of a non-metric multidimensional scaling (NMDS) analysis of Bray–Curtis dissimilarities in the OTUs community matrix. The main microbial predictors for the cycling of multi-nutrients in soil were identified by a classification random forest (RF) analysis [78]. In these RF models, microbial alpha- and beta-diversity indices served as predictors for the soil multi-nutrient cycling index. To estimate the importance of these diversity indices, we used percentage increases in the MSE (mean squared error) of variables [79]: higher MSE% values imply more important variables [79].

Significance of the models and cross-validated *R*^2^ values were assessed with 5000 permutations of the response variable, by using the “A3” package. Similarly, the significance of each predictor on the response variables was assessed with the “rfPermute” package. We also applied an RF analysis to estimate the importance of dominant phyla (those > 5% of the total community) for explaining the soil properties. A multiple regression model with variance decomposition analysis was used to validate the RF analysis outcome by using the lm and calc.relimp function in the “relaimpo” package.

## Additional file

Additional file 1: Supporting Information for Soil microbiomes with distinct assemblies through vertical soil profiles drive the cycling of multiple nutrients in reforested ecosystems, including: Supporting information Results, **Table S1-S4** and **Figure S1-S11**. (DOCX 4693 kb)
