# Supplementary material for: Soil microbiomes with distinct assemblies through vertical soil profiles drive the cycling of multiple nutrients in reforested ecosystems
Source: Microbiome. 2018 Aug 21;6:146. doi: 10.1186/s40168-018-0526-0 (PMC6104017; doi:10.1186/s40168-018-0526-0)

**Supporting Information for**

**Soil microbiomes with distinct assemblies through vertical soil profiles drive the cycling of multiple nutrients in reforested ecosystems**

**Running title:** Soil microbiome drive subsurface nutrients cycling

Shuo Jiao^1, 2^, Weimin Chen^1^*, Jieli Wang^1^, Nini Du^1^, Qiaoping Li^1^, Gehong Wei^1^*

^1^ *State Key Laboratory of Crop Stress Biology in Arid Areas*, *College of Life Sciences, Northwest A&F University*, *Yangling Shaanxi 712100*, *P*. *R*. *China*;

^2^ *College of Urban and Environmental Sciences*, *Peking University*, *Beijing 100871*, *P*. *R*. *China*

*Correspondence: Weimin Chen or Gehong Wei, State Key Laboratory of Crop Stress Biology in Arid Areas, College of Life Sciences, Northwest A&F University, Yangling Shaanxi 712100, P. R. China.

E-mail: chenwm029@nwsuaf.edu.cn or weigehong@nwsuaf.edu.cn; Tel.: +86-29-87091175; Fax: +86-29-87091175

***Supporting information***

***Results***

*Temporal and spatial distribution patterns of microbial taxa at fine scale*

The distribution of dominant phyla showed that the Gemmatimonadetes, Euryarchaeota, Ascomycota, and Zygomycota dominated the arable land, whereas the Betaproteobacteria, Deltaproteobacteria, Acidobacteria, Nitrospirae, and Basidiomycota were dominant in the reforested soils (**Figure S6**). In the course of reforestation, the relative abundance of Nitrospirae, Thermotogae, Euryarchaeota, and Basidiomycota all decreased, whereas relative abundance of Alphaproteobacteria, Actinobacteria, and Planctomycetes increased **(Figure S7**).

Next, significant taxonomic differences among reforestation years were examined with a Canonical discriminant analysis based on genera with a relative abundance of >0.5% (**Figure S8**). In the 10-year reforested soil, GAL15, *Arthrobacter*, *Lysurus*, *Monographella*, and *Volutella* were dominant. In the 20-year reforested soil, *Bradyrhizobium*, *Gaiella*, *Microidium*, *Clathrus*, and *Crustoderma* were dominant. In the 30-year reforested soil, *Pirellula*, *Acinetobacter*, *Chryseolinea*, *Entotheonella*, *Fusarium*, *Mortierella*, and *Trichoderma* were dominant. This analysis was not conducted for archaea, because the majority of archaeal operational taxonomic units (OTUs) could not be classified to the genus level.

Further, we explored the vertical spatial distribution of the dominant phyla in arable land and reforested soils (**Figures S9 and S10**). Several phyla showed a similar vertical distribution in the arable land and reforested soils. For example, as soil depth increased, Deltaproteobacteria decreased through the profiles; Nitrospirae, Thermotogae, and Euryarchaeota increased in the superficial layers; and Bacteroidetes decreased in the superficial layers. However, most of the phyla showed different vertical distribution patterns between the arable land and reforested soils. Alphaproteobacteria, Chloroflexi, and Gemmatimonadetes significantly varied with soil depth in the arable land, but they did not change significantly in the reforested soils; this pattern was reversed for Verrucomicrobia and Zygomycota. The Betaproteobacteria, Actinobacteria, and Basidiomycota also showed contrasting trends between the arable land and reforested soils.

**Table S1** Coordinates of the 43 sampling sites corresponding to the chronosequence of reforestation sites in the ex-arable land.

| ***Topological feature*** | ***Longitude*** | ***Latitude*** | ***Altitude*** |
| --- | --- | --- | --- |
| Arable land | E107°55’50’’ | N34°33’18’’ | 1058 |
| 10-year forest | E107°55’33’’ | N34°33’24’’ | 1160 |
| 20-year-Forest | E107°55’32’’ | N34°33’42’’ | 1138 |
| 30-year-Forest | E107°55’17’’ | N34°33’41’’ | 1146 |

**Table S2** ANOSIM and permutational MANOVA analyses of microbial beta-diversities between superficial and deep layers, in the arable land and reforested soils respectively.

|  |  | ***ANOSIM*** | |  | ***ADONIS*** | |
| --- | --- | --- | --- | --- | --- | --- |
|  |  | ***R*** | ***P*** |  | ***R^2^*** | ***P*** |
| Bacteria | Arable land | 0.3044 | 0.001 |  | 0.1615 | 0.001 |
|  | 10-year forest | 0.1858 | 0.001 |  | 0.0837 | 0.001 |
|  | 20-year Forest | 0.0364 | 0.0027 |  | 0.0251 | 0.001 |
|  | 30-year Forest | 0.0875 | 0.001 |  | 0.0359 | 0.001 |
| Archaea | Arable land | 0.1799 | 0.005 |  | 0.1321 | 0.001 |
|  | 10-year forest | 0.4188 | 0.001 |  | 0.2042 | 0.001 |
|  | 20-year Forest | 0.1745 | 0.001 |  | 0.0854 | 0.001 |
|  | 30-year Forest | 0.2243 | 0.002 |  | 0.1057 | 0.001 |
| Fungi | Arable land | 0.0191 | 0.235 |  | 0.0402 | 0.221 |
|  | 10-year forest | 0.0473 | 0.005 |  | 0.0313 | 0.007 |
|  | 20-year Forest | 0.0426 | 0.014 |  | 0.0280 | 0.01 |
|  | 30-year Forest | 0.1395 | 0.001 |  | 0.0587 | 0.001 |

**Table S3** Variation explained by microbial alpha- and beta-diversity indices in regression models of the soil multi-nutrient cycling index, for the whole profiles, and superficial and deep layers separately.

| ***Microbial diversity index*** | | ***Whole*** | ***Superficial*** | ***Deep*** |
| --- | --- | --- | --- | --- |
| Bacteria | Alpha-Shannon |  |  |  |
|  | Alpha-Richness | 4.52% | 3.27% |  |
|  | Beta-NMDS1 | 10.68% | 5.67% | 29.19% |
|  | Beta-NMDS2 |  | 1.34% | 18.45% |
| Archaea | Alpha-Shannon | 4.43% | 6.07% |  |
|  | Alpha-Richness | 1.74% |  | 4.61% |
|  | Beta-NMDS1 | 2.36% | 11.12% |  |
|  | Beta-NMDS2 | 2.99% | 2.03% |  |
| Fungi | Alpha-Shannon | 1.31% | 2.34% |  |
|  | Alpha-Richness | 2.04% | 5.43% |  |
|  | Beta-NMDS1 | 6.71% | 4.75% | 6.30% |
|  | Beta-NMDS2 |  |  | 0.99% |
| **Total** | | **36.76%** | **42.02%** | **59.55%** |

NA, not statistically significant (*P*>0.05)

**Table S4** Variation explained by dominant phyla (>5% of total community) in regression models of soil environmental variables.

| ***Dominant phyla*** | | ***Organic matter*** | ***Nitrate-N*** | ***Total P*** | ***Available P*** | ***Available K*** | ***pH*** |
| --- | --- | --- | --- | --- | --- | --- | --- |
| Bacteria | Alphaproteobacteria |  |  | 27.65% | 9.94% | 2.93% |  |
|  | Betaproteobacteria |  |  | 10.46% |  |  |  |
|  | Deltaproteobacteria |  |  | 1.49% | 4.56% | 1.91% |  |
|  | Actinobacteria | 0.90% | 2.07% | 9.13% | 6.24% |  |  |
|  | Acidobacteria | 4.82% | 10.06% |  | 4.86% | 7.30% | 5.87% |
|  | Chloroflexi |  |  | 14.13% |  | 0.85% | 1.55% |
|  | Nitrospirae |  | 1.53% | 3.42% | 3.83% | 1.37% |  |
|  | Thermotogae |  |  | 9.16% | 11.78% | 3.20% | 5.58% |
| Archaea | Thaumarchaeota | 6.11% | 4.32% |  | 0.55% |  | 5.85% |
|  | Euryarchaeota | 2.60% | 5.57% | 0.75% | 10.63% | 4.45% | 6.07% |
| Fungi | Ascomycota |  |  |  |  | 0.32% |  |
|  | Basidiomycota |  |  |  |  | 0.33% |  |
|  | Zygomycota | 1.79% | 5.13% |  |  | 0.81% |  |
| **Total** | | **16.21%** | **28.68%** | **76.18%** | **52.39%** | **24.92%** | **23.47%** |

NA, not statistically significant (*P*>0.05)

**Figure S1** (A) Variation in soil properties during reforestation of the ex-arable land estimated by linear mixed-effects models, with samples from the same tree (reforested soils) or the same core (arable land) set as random effects. Boxplots that do not share a letter are significantly different (*P* < 0.05). (B) Variation in soil properties with depth. Linear least-squares regression relationships between soil depth and soil properties were estimated. The adjust R^2^ was used to select determine the models were fitted with the whole depths or with superficial and deep layers separately. The lines denote the least-squares linear regressions across soil depth, with their 95% confidence intervals (gray-shaded areas). “w”, slope of the fitting line; *, *P* < 0.05; **, *P* < 0.01; ***, *P* < 0.001.


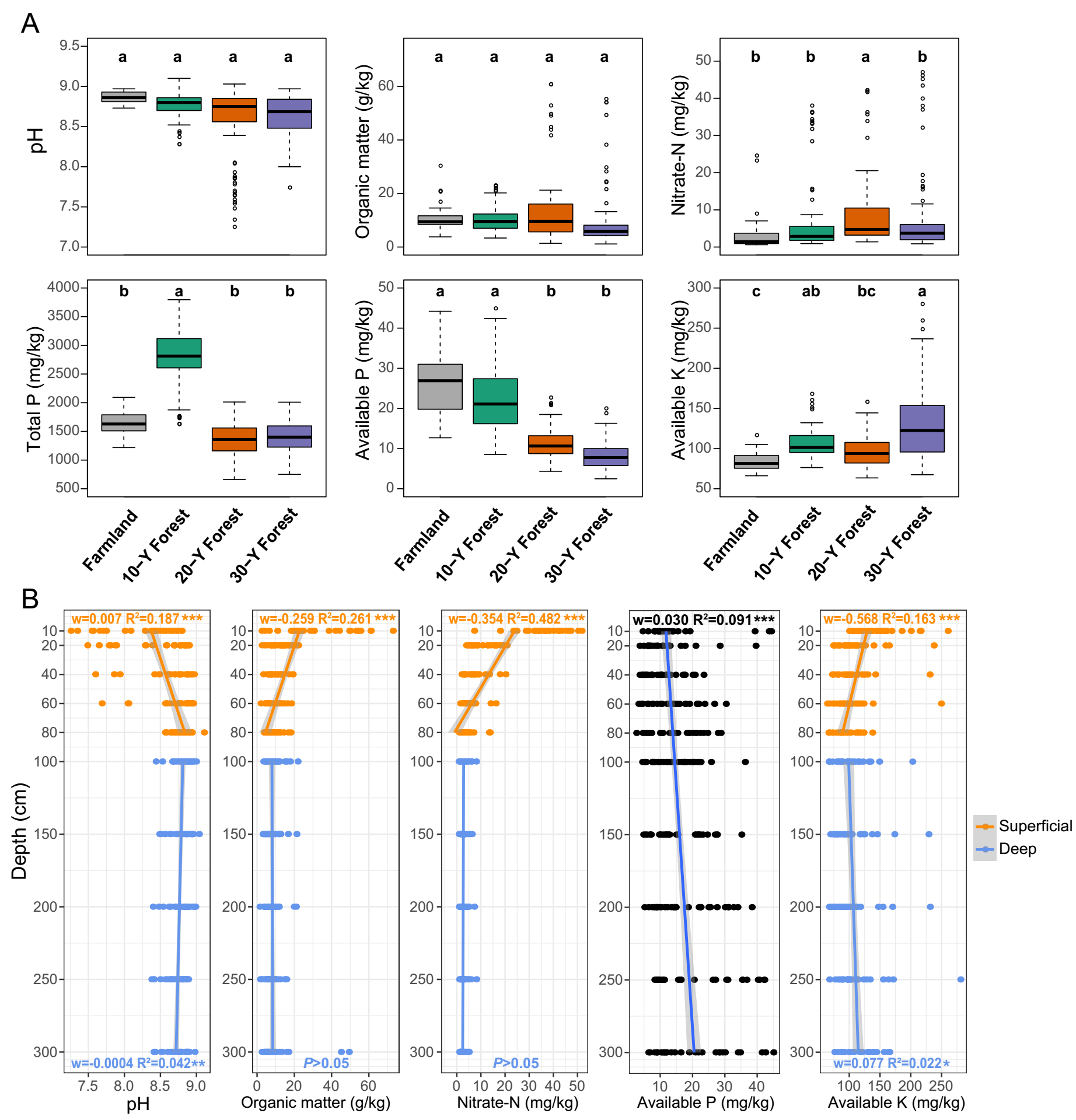


**Figure S2** Variation in available phosphorus with depth in arable land (“Farmland”) and reforested soils (“Forest”). Linear least-squares regression relationships between soil depth and physicochemical properties were estimated. The adjust R^2^ was used to determine whether the models were fitted with whole depths or with superficial and deep layers separately. The lines denote the least-squares linear regressions across soil depth, with their 95% confidence intervals (gray-shaded areas). “w”, slope of the fitting line; **, *P* < 0.01; ***, *P* < 0.001.


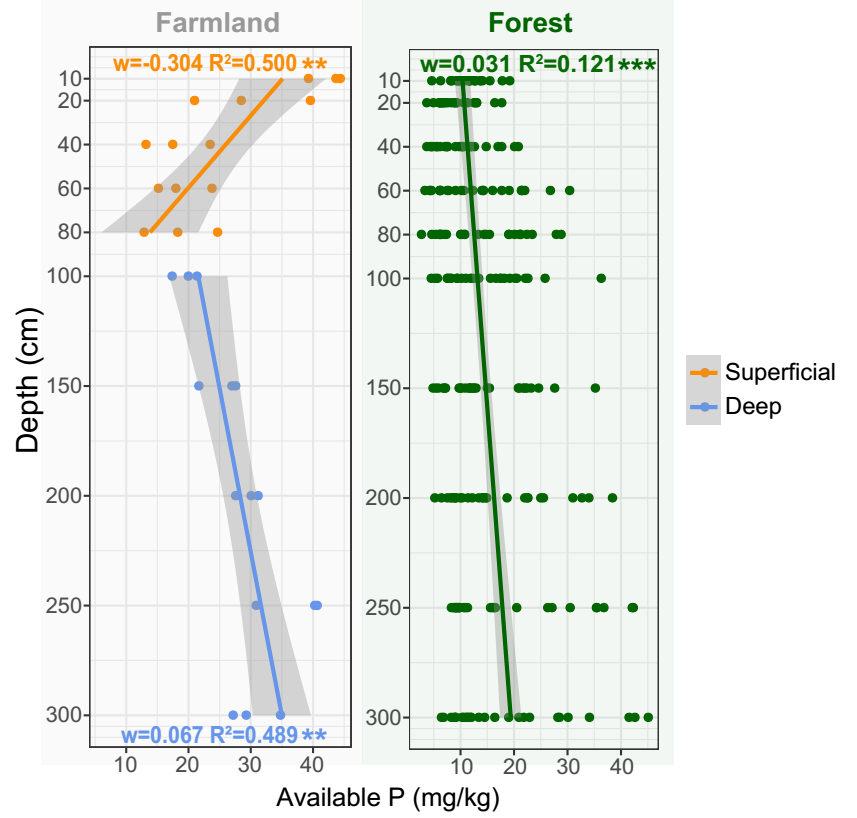


**Figure S3** Variation in soil properties between superficial and deep layers in arable land and reforested soils. Significances of these differences was evaluated based on linear mixed-effects models, with samples from the same tree (reforested soils) or the same core (arable land) set as random effects. *, *P* < 0.05.


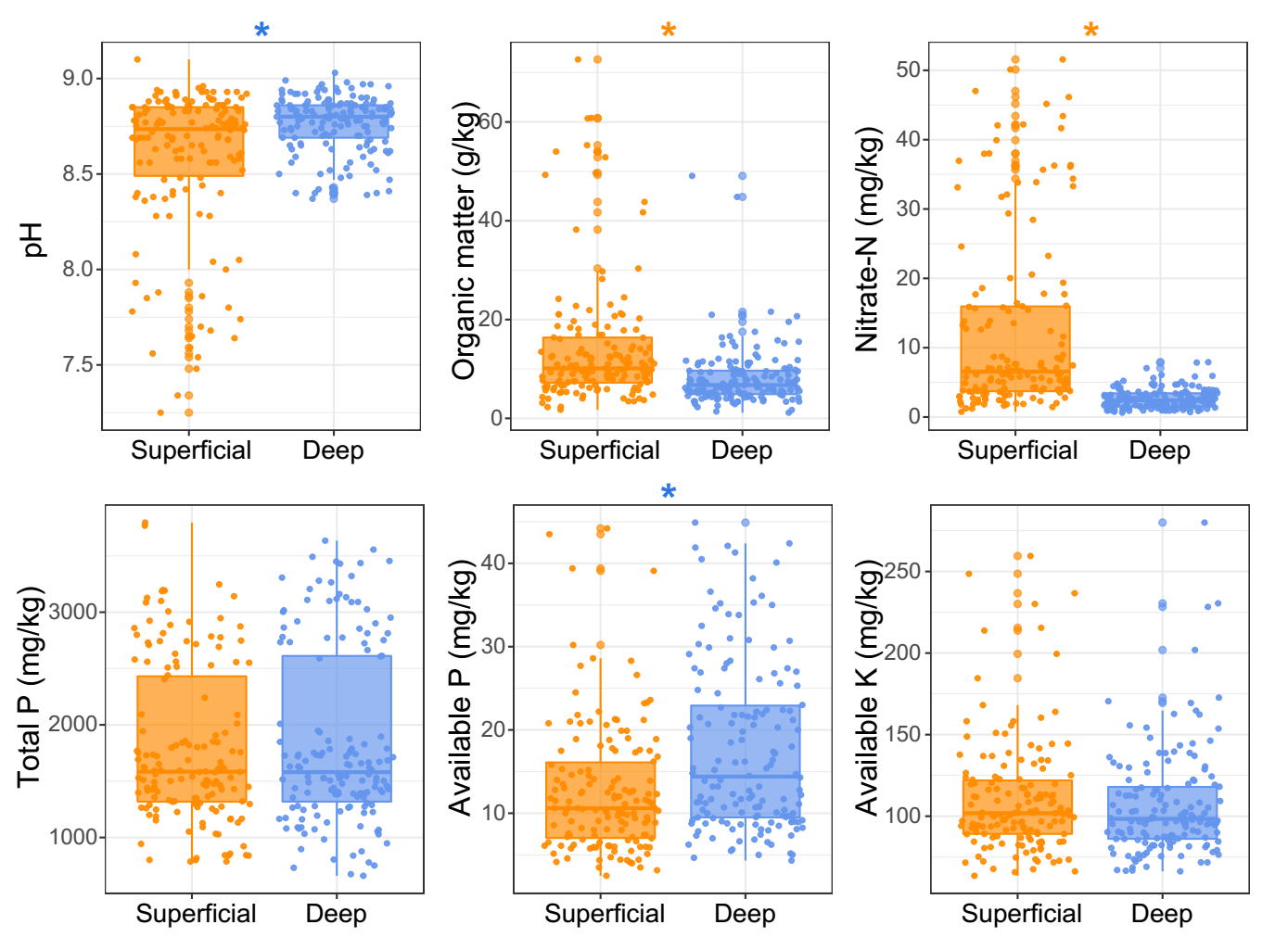


**Figure S4** Variation in alpha-diversity of soil bacteria, archaea, and fungi between soil depths in arable land (“Farmland”) and reforested soils (“Forest”). The lines denote the least-squares linear regressions across soil depths. The lines denote the least-squares linear regressions across soil depth, with their 95% confidence intervals (gray-shaded areas). “w”, slope of the fitting line; *, *P* < 0.05; **, *P* < 0.01; ***, *P* < 0.001.


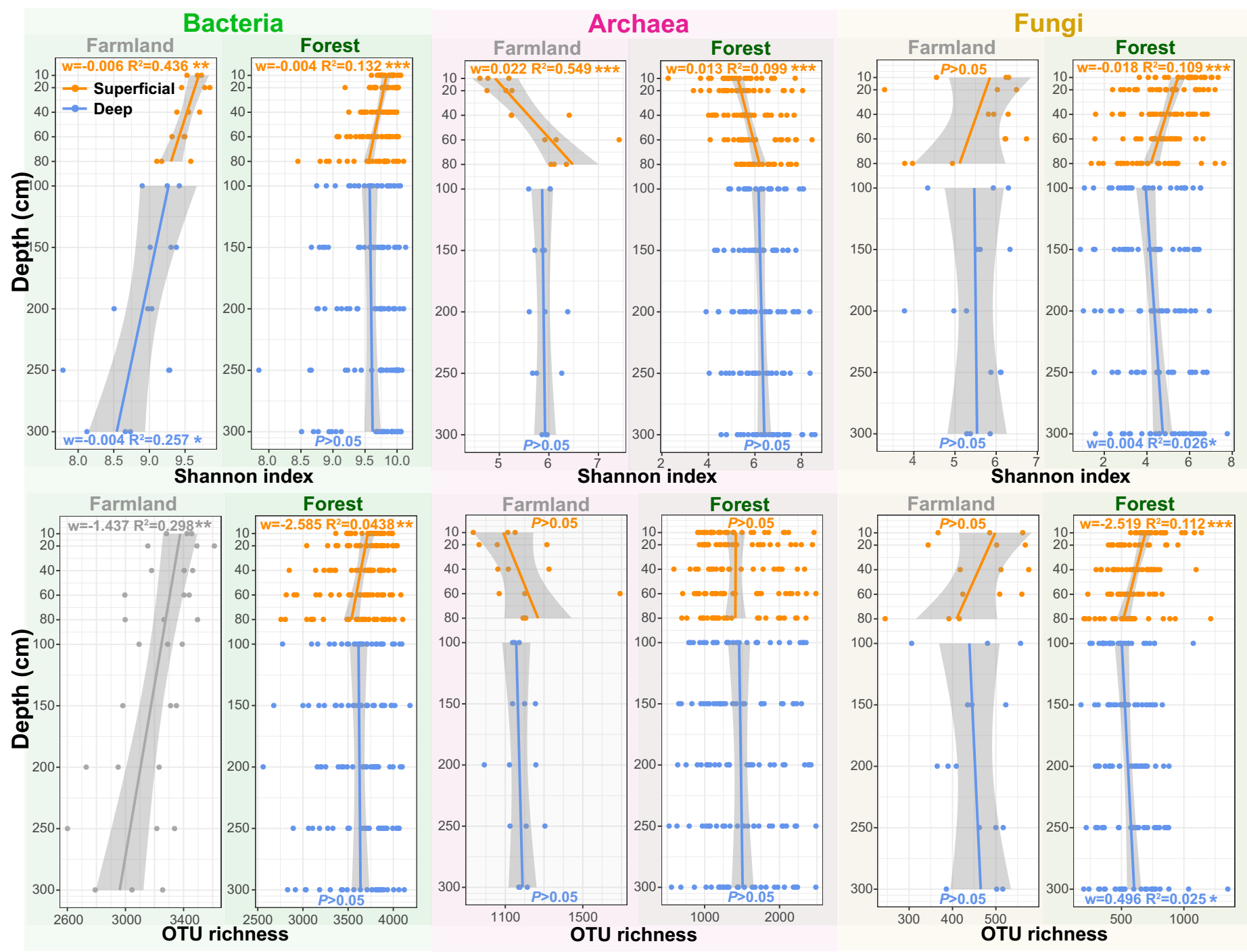


**Figure S5** Variation in alpha-diversity of soil bacteria, archaea, and fungi between superficial and deep layers in arable land (“Farmland”) and reforested soils (“Forest”). Significances of these differences was evaluated by linear mixed-effects models, with samples from the same tree (reforested soils) or the same core (arable land) set as random effects. *, *P* < 0.05.


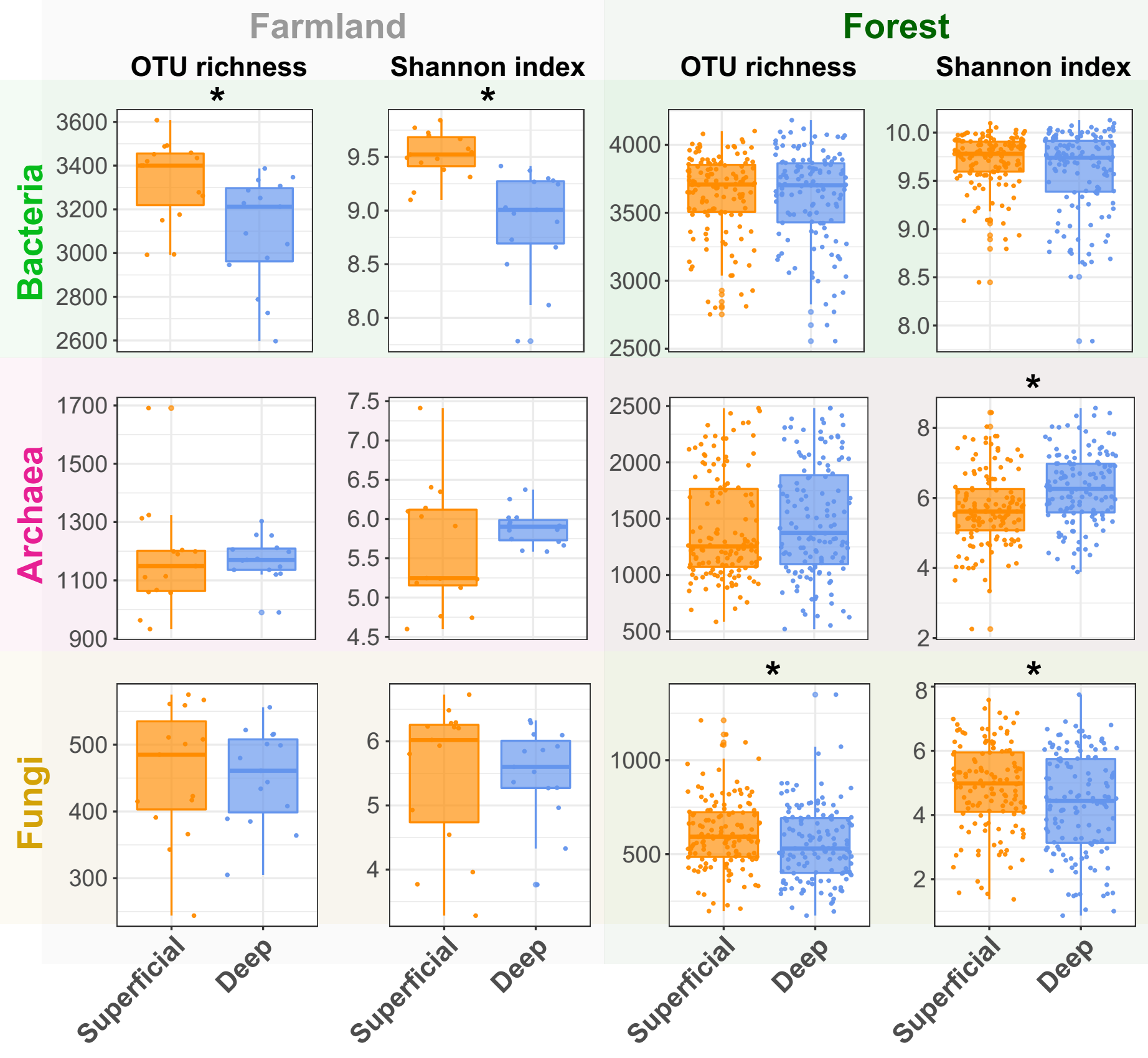


**Figure S6** Variation in the relative abundance of dominant phyla (>1% of total community) between arable land (“Farmland”) and reforested soils (“Forest”). All show significant group differences based on linear mixed-effects models, with samples from the same tree (reforested soils) or the same core (arable land) set as random effects (*P* < 0.05).


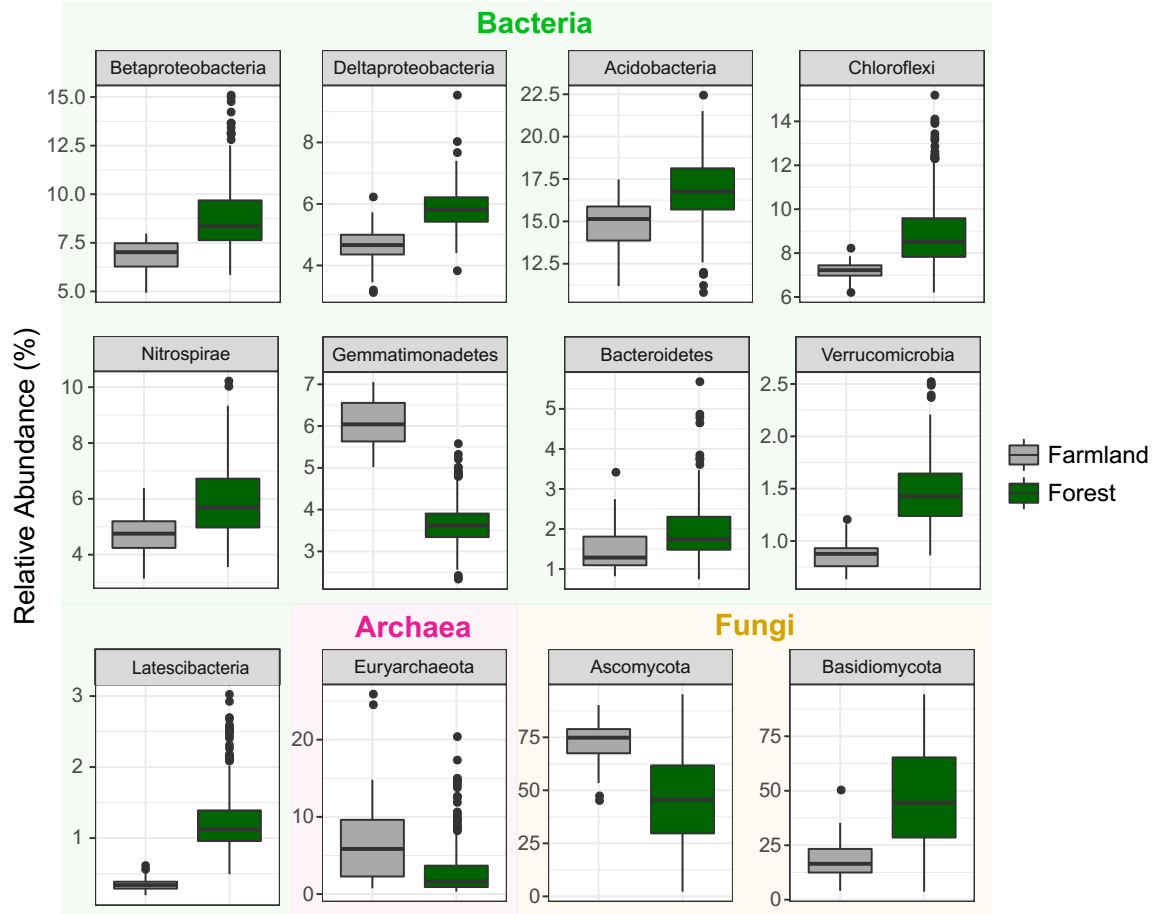


**Figure S7** Variation in the relative abundance of dominant phyla (>1% of total community) in soils among different reforestation years. All show significant group differences based on linear mixed-effects models, with samples from the same tree set as random effects (*P* < 0.05).


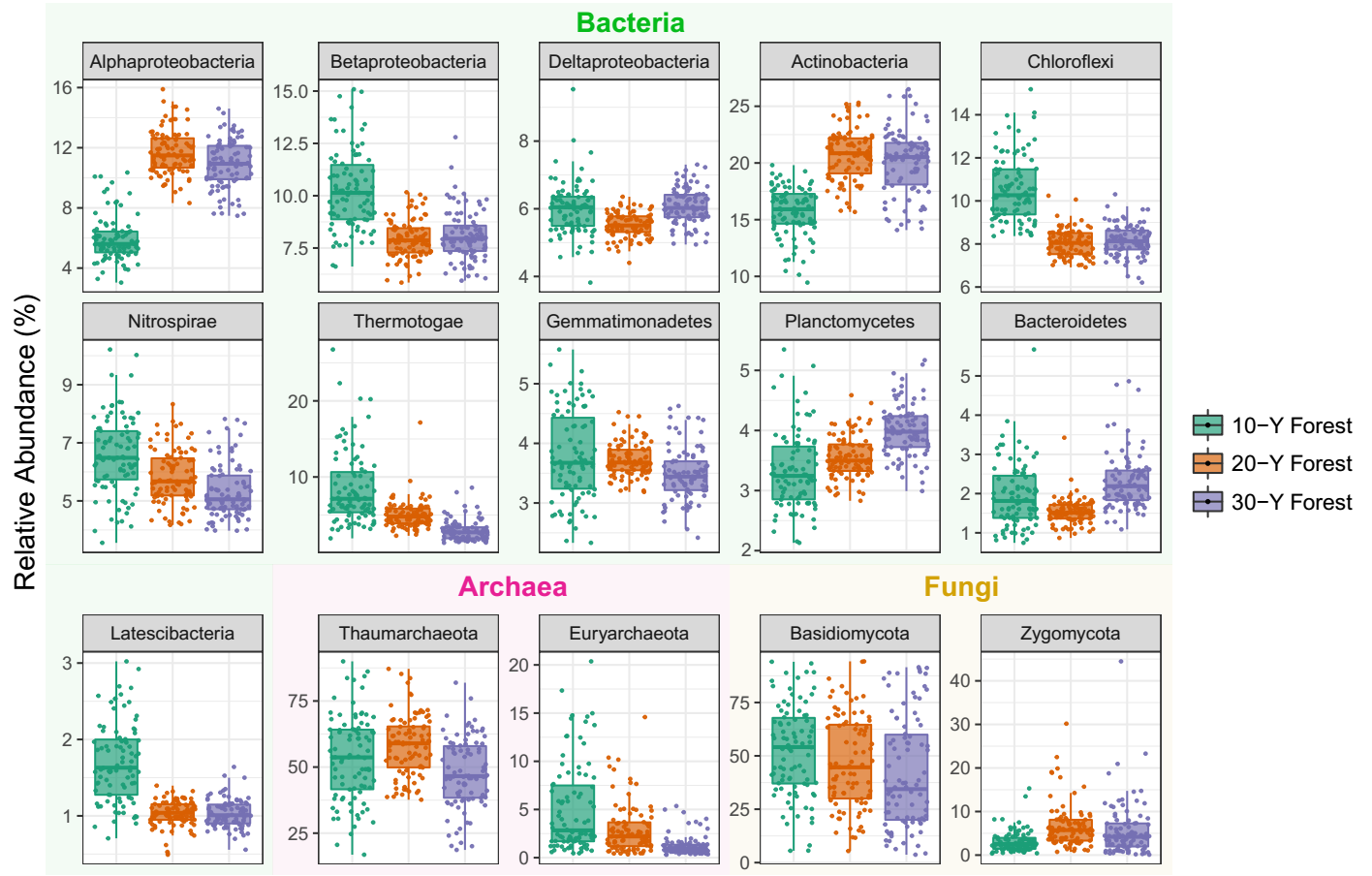


**Figure S8** Canonical discriminant analysis comparing reforestation years against bacterial (A) and fungal (B) taxa loadings based on genera with relative abundance > 0.1%. Arrows represent the degree of correlation between each taxon and each treatment as a measure of the predictive discrimination of each treatment. Circles represent the canonical group means and 95% confidence interval for each class.


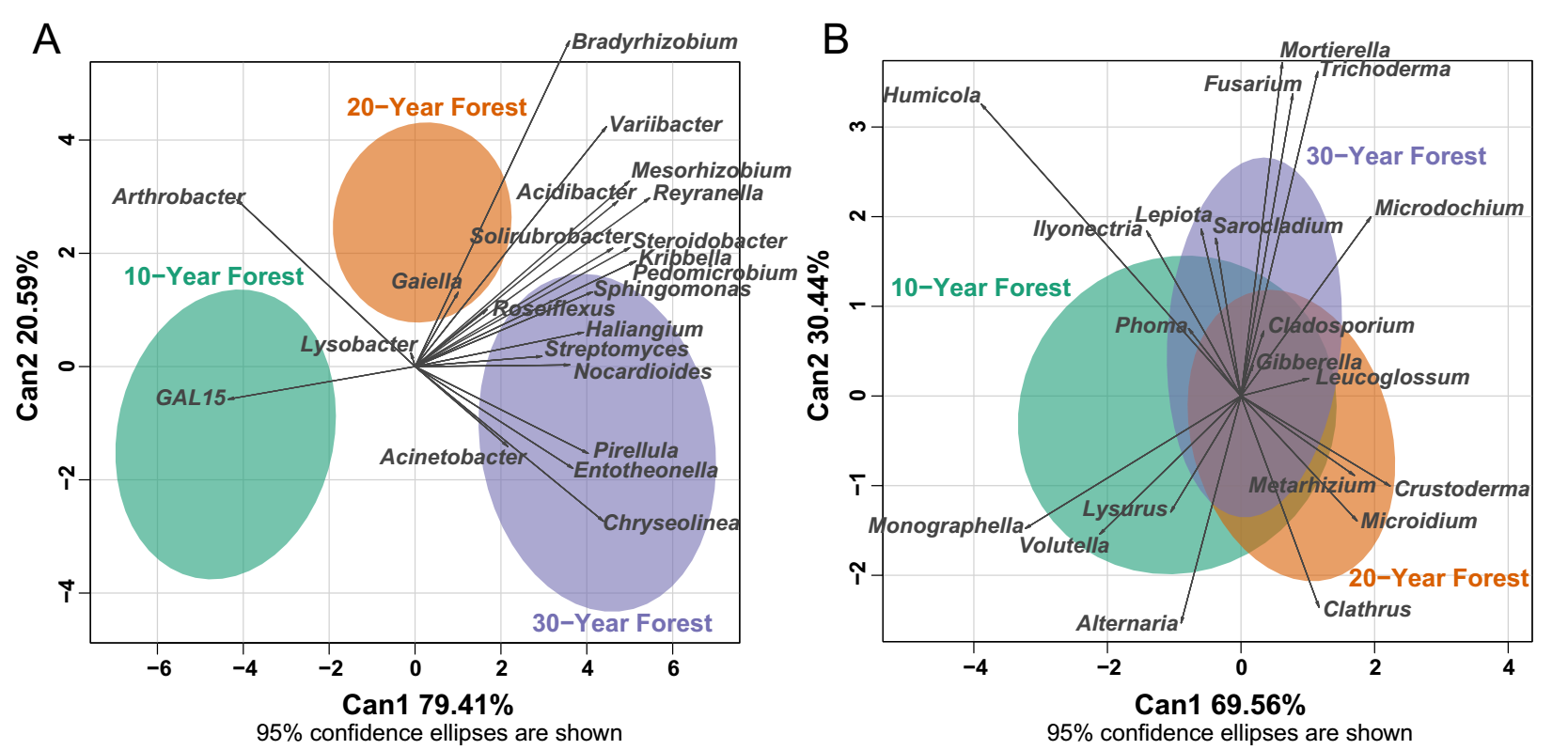


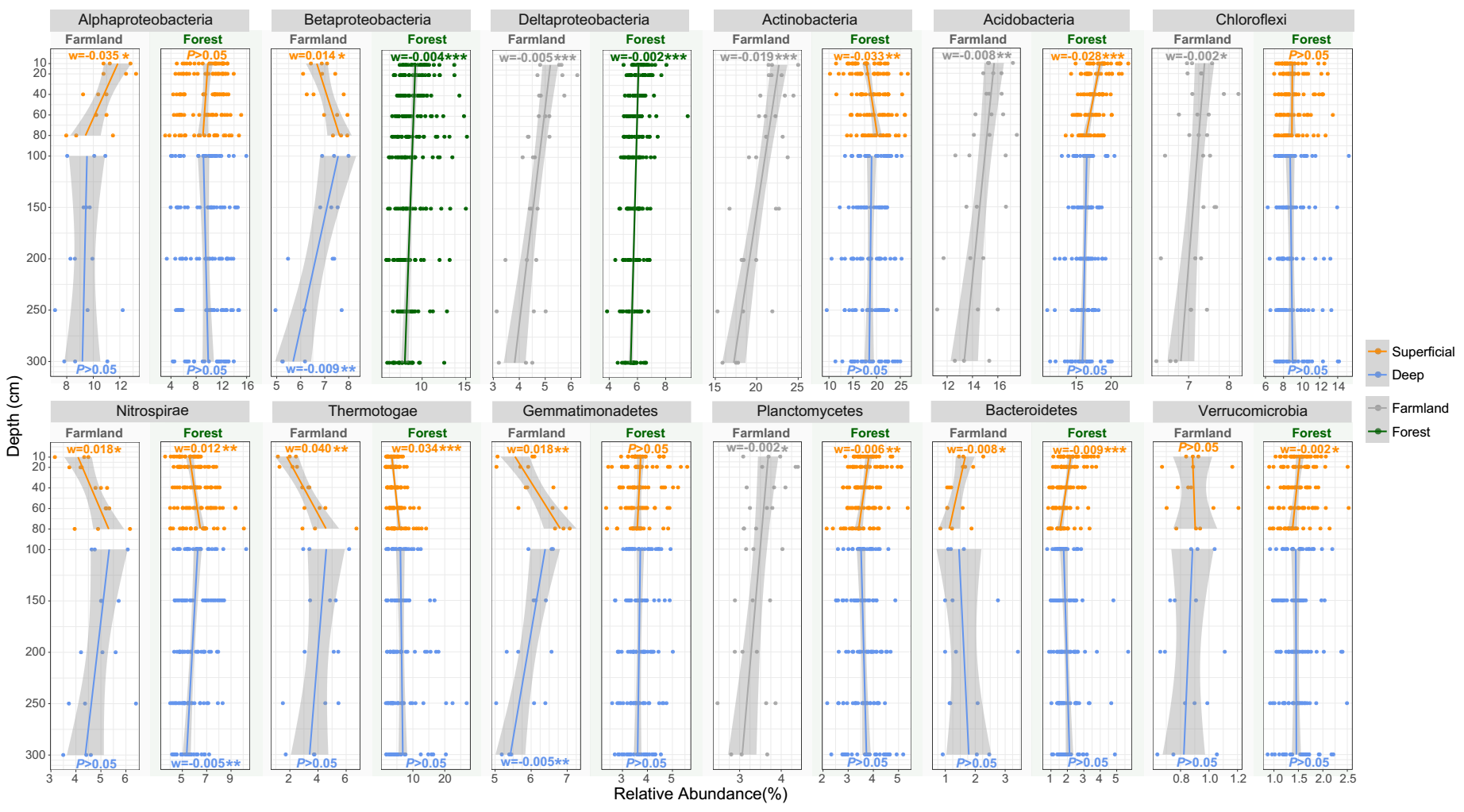
**Figure S9** Vertical spatial variation in the dominant bacterial phyla with relative abundance > 1% through vertical soil profiles in arable land (“Farmland”) and reforested soils (“Forest”). Lines denote least-squares linear regressions across soil depths. The lines denote the least-squares linear regressions across soil depth, with their 95% confidence intervals (gray-shaded areas). “w”, slope of the fitting line; “w”, slope of the fitting line; *, *P* < 0.05; **, *P* < 0.01; ***, *P* < 0.001.

**Figure S10** Vertical spatial variation in the dominant fungal and archaeal phyla with relative abundance > 1% through vertical soil profiles in arable land (“Farmland”) and reforested soils (“Forest”). Lines denote least-squares linear regressions across soil depths. The lines denote the least-squares linear regressions across soil depth, with their 95% confidence intervals (gray-shaded areas). “w”, slope of the fitting line; *, *P* < 0.05; **, *P* < 0.01; ***, *P* < 0.001.


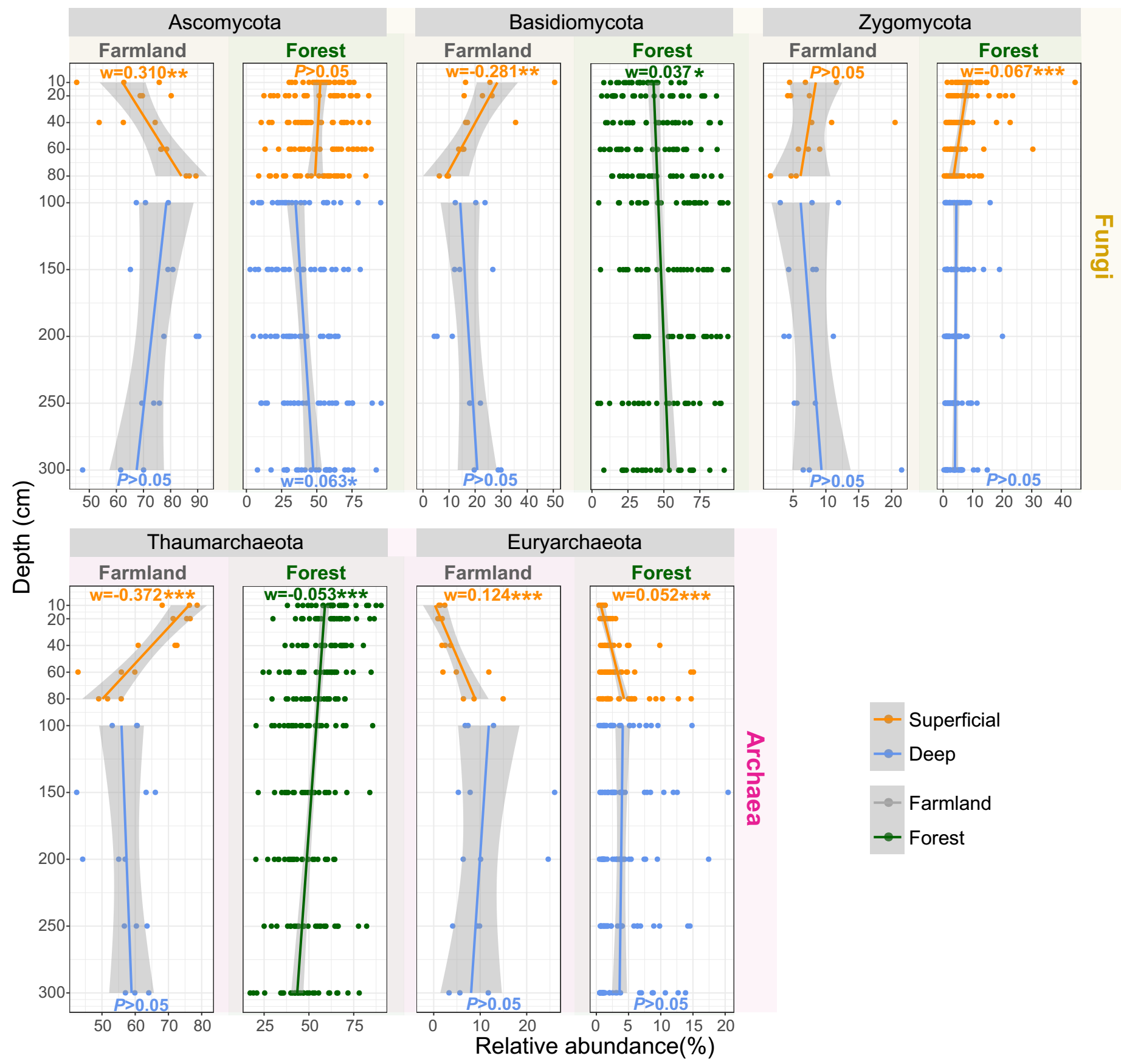


**Figure S11** Taxonomic distribution of fungal taxa responsible for community differentiation among different radii to the tree at each soil depth. The most abundant six genera are displayed in Barplots. Ternary plots show the distributions of these differentiation taxa. Each circle represents one OTU. The size of each circle represents its relative abundance. The position of each circle is determined by the contribution of the indicated compartments to the total relative abundance. The color of the circles marks the OTUs significantly enriched among different radiations to plant (FDR<0.01). The numbers of differential OTUs are displayed at the vertex of the ternary plots.


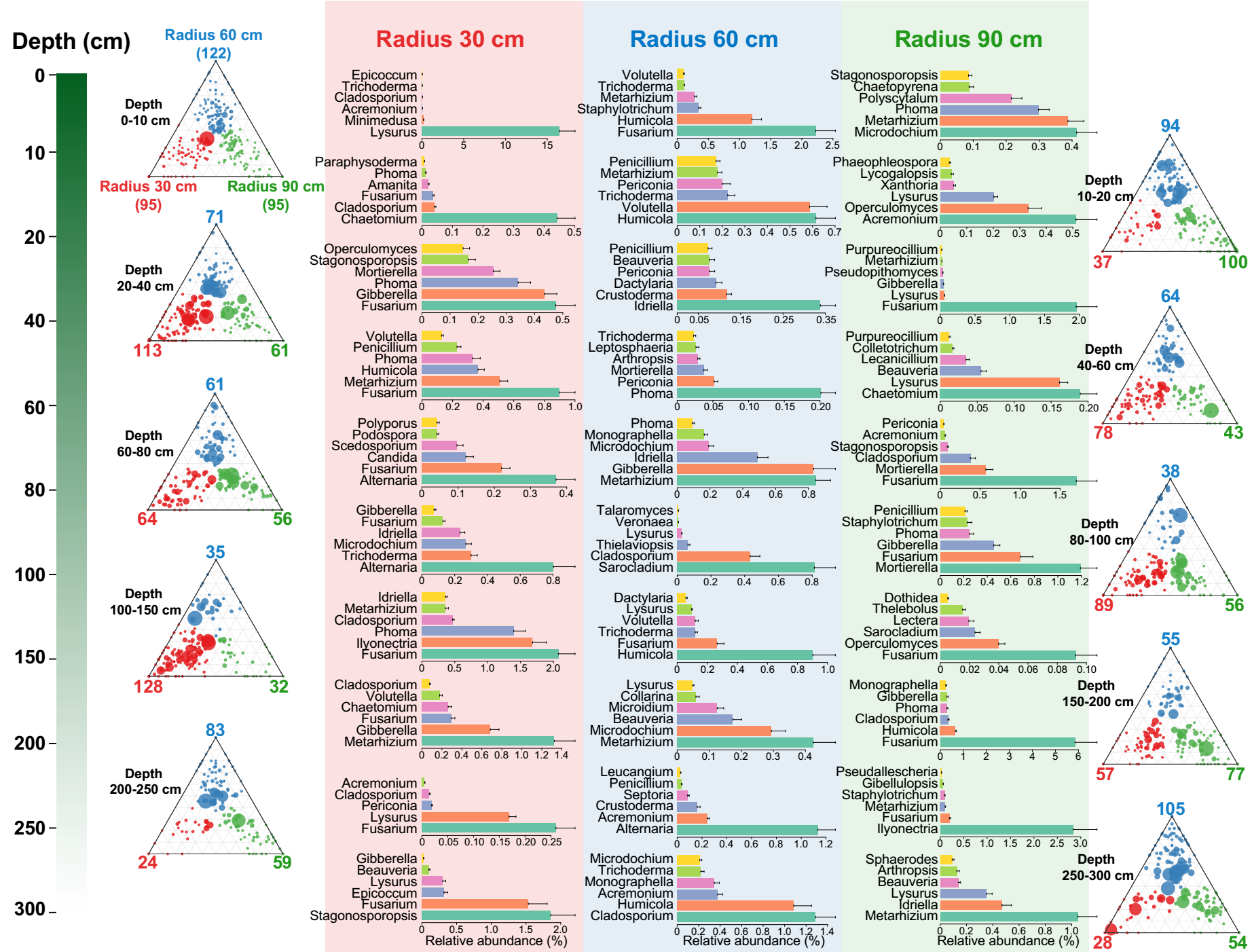

Supplement: Supplementary file 1 — Supporting Information for Soil microbiomes with distinct assemblies through vertical soil profiles drive the cycling of multiple nutrients in reforested ecosystems, including: Supporting information Results, Table S1-S4 and Figure S1-S11. (DOCX 4693 kb) [file 40168_2018_526_MOESM1_ESM.docx]
